# Environmental Adaptation in *Nostoc sphaeroides*: Spherical Multicellularity, Molecular Signalling and Implications for Sustainable Cultivation

**DOI:** 10.3390/plants15182856

**Published:** 2026-09-18

**Authors:** Jin-Long Shang, Lu-Yao Shi, Jun-Ying Lv, Qiao-Chu Zhou, Jin-Yan Guan

**Affiliations:** 1 Xinjiang Key Laboratory of Special Species Conservation and Regulatory Biology, College of Life Science, Xinjiang Normal University, Urumqi 830054, China; 2 Desert Algae Research Institute, Xinjiang Normal University, Urumqi 830054, China; 3 College of Chemistry and Chemical Engineering, Xinjiang Normal University, Urumqi 830054, China

**Keywords:** cyanobacteria, cyanosphere, developmental multicellularity, environmental sensing, extracellular polymeric substances, paddy fields, ultraviolet acclimation

## Abstract

*Nostoc sphaeroides* is a diazotrophic cyanobacterium that forms macroscopic spherical colonies in seasonally flooded paddy fields. Studies of its environmental physiology, colony development and cultivation have largely proceeded independently. This review integrates strain-resolved genomic, transcriptomic, physiological and ecological evidence to examine spherical multicellularity as a possible adaptation to a variable habitat. Developmental transitions between motile hormogonia and vegetative, heterocyst-forming filaments link dispersal with establishment, while an extracellular polymeric substance (EPS) matrix promotes water retention, ion binding and formation of an associated cyanosphere. Colony enlargement may improve persistence but also produces gradients of light, gases and nutrients that can limit carbon acquisition. Visible light, low-dose ultraviolet radiation, nitrogen form, phosphate, calcium, hydration and agrochemicals influence photosynthetic electron flow, carbon-concentrating mechanisms, repair and developmental investment. The NsHik33-NsRpaB pathway provides a causal connection between environmental sensing and ultraviolet acclimation, whereas many proposed colony-level functions remain supported mainly by omics data or evidence from model cyanobacteria. Important research priorities include spatial chemical mapping, defined cyanosphere communities, multiplex genetics and experiments under fluctuating environmental conditions. Together, the evidence identifies *N. sphaeroides* as a promising organism for studying how cyanobacterial developmental multicellularity promotes persistence in variable environments and may inform cultivation practices.

## 1. Introduction

Edible terrestrial and freshwater cyanobacteria lie at the intersection of microbial ecology, photosynthetic stress physiology and food production [1,2]. *Nostoc sphaeroides* Kützing (hereafter *N. sphaeroides*), known in China as Ge-Xian-Mi or rice-field *Nostoc*, is notable for a developmental programme that produces hydrated spherical colonies ranging from millimetres to centimetres in diameter. Natural populations occur in shallow paddy-field waters and at the soil-water interface, where canopy shade, sunflecks, flooding, drainage, nutrient additions and agricultural chemicals vary over hours to seasons [3,4]. These conditions raise a central question in environmental microbiology: how does a cyanobacterium translate temporal environmental variation into a stable multicellular phenotype?

Previous reviews have focused mainly on composition, food processing, bioactive polysaccharides or production technology [5,6,7]. These perspectives establish the value of the organism but do not fully connect habitat, developmental transitions, matrix formation, microbial partners and regulatory mechanisms. Colony size, hydration, nitrogen status, pigment content and extracellular polymer production are not independent traits; they arise from interacting processes involving dispersal, cell differentiation, photosynthetic energy balance, carbon acquisition and investment in a shared extracellular matrix.

A mechanistic synthesis is now possible because *N. sphaeroides* has a complete multipartite genome, stage-resolved transcriptomes, ultraviolet-focused proteomics and methods for targeted genetic manipulation [8,9,10]. These advances allow field observations to be examined through causal experiments more readily than was possible a decade ago. Nevertheless, genomic divergence among strains identified as *N. sphaeroides* and the frequent use of non-axenic cultures require careful attention to strain identity and community composition.

We organise the evidence across four biological scales: paddy-field variation, the colony microenvironment, differentiated cells and regulatory mechanisms. Observations made directly in *N. sphaeroides* are distinguished from interpretations based on other filamentous cyanobacteria, and important inferences are considered alongside experiments that could test them. We argue that the sphere is more than passive packaging: it is a developmentally constructed microbial habitat whose benefits, including hydration, buffering and persistence, are inseparable from the costs of self-shading and limited diffusion. This perspective clarifies ecological hypotheses and suggests how culture studies can improve reproducibility.

## 2. Strain Variation and the Fluctuating Paddy-Field Habitat

### 2.1. Taxonomic Identity Is an Experimental Variable

The name *N. sphaeroides* is often used as though it described a uniform biological entity, but comparative genomics indicates substantial diversity. Two isolates assigned to the species can share 100% identity in the 16S rRNA gene while showing only 93.48% average nucleotide identity, below the 95–96% range commonly used to delimit prokaryotic species [11,12]. Such divergence may affect regulatory repertoires, plasmid content, nutrient acquisition, natural-product potential and interactions with heterotrophic bacteria. Findings from strain CCNUC1, CHAB2801 or commercial material should therefore not be generalised without qualification.

At a minimum, studies should report a stable strain identifier, source locality, axenic or non-axenic status, genome-based placement, culture history and the colony size distribution used in each experiment. Medium composition, photon spectrum and flux, temperature, mixing and developmental stage are also important because colony morphology can change during serial propagation. A reference collection linking complete genomes with cryopreserved vouchers and well-described phenotypes would help distinguish conserved mechanisms from lineage-specific features.

### 2.2. Paddy-Field Conditions Vary in Sequence, Not in Isolation

Field surveys in Hefeng County associated productive *N. sphaeroides* habitats with mildly acidic soils, available phosphorus, shallow water and low-intensity management [3]. A later survey recorded acidic water (pH 6.30) in a field where the organism was absent; this field also had the lowest water conductivity and soil total and available phosphorus among the sites compared [4]. These observations argue against a single-factor explanation based on preferred pH. Establishment is more likely to depend on interactions among mineral supply, ionic strength, hydrology, propagule availability, biological competition and disturbance history.

The order of environmental events may matter as much as their average intensity. Flooding can increase turbidity and reduce scalar irradiance, followed by sunflecks as the canopy or water column changes. Drainage concentrates ions, increases temperature variation and causes water loss. Fertiliser and pesticide applications create short chemical pulses, while colony optics and diffusion change continuously as the sphere enlarges. Constant-light, single-factor cultures reveal physiological capacity but do not reproduce these environmental sequences. Factorial microcosms with defined histories of light, temperature and wetting would more closely reflect conditions in the field.

### 2.3. Development Couples Dispersal to Establishment

**Figure 1:**
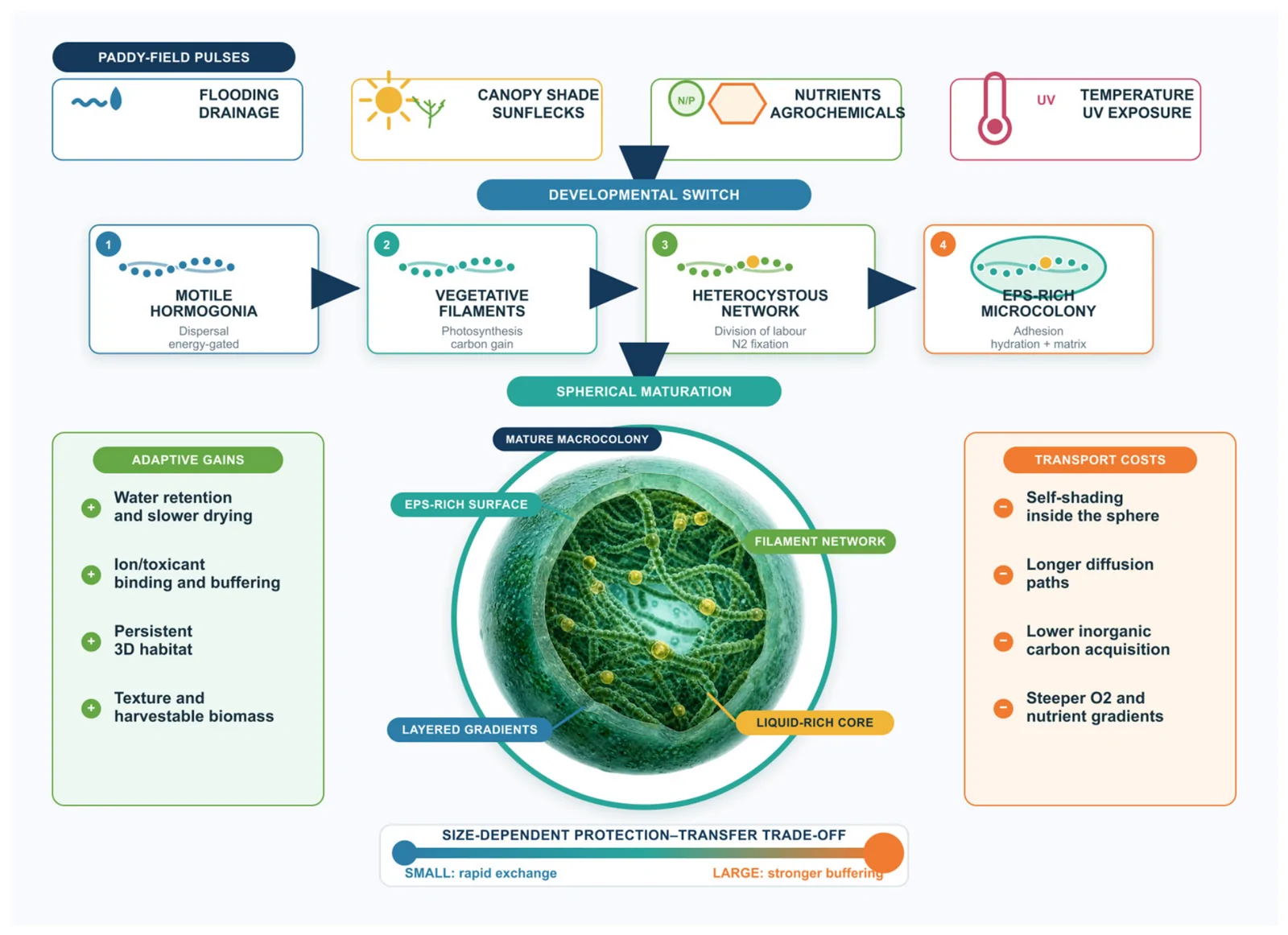
Developmental transitions and niche construction in *N. sphaeroides*. Paddy-field variation influences transitions from dispersive hormogonia to vegetative filaments, heterocyst-containing networks, extracellular-polymer-rich microcolonies and mature spherical macrocolonies. Enlargement may increase persistence, water retention and buffering while reducing light penetration and the exchange of gases and solutes. Abbreviations: Ci, inorganic carbon; EPS, extracellular polymeric substances; N, nitrogen; UV, ultraviolet radiation.

The life cycle links motile hormogonia with sessile, nitrogen-fixing colonial growth (Figure 1). In *N. sphaeroides*, hormogonium differentiation is strongly influenced by light and energy status. The reported optimum irradiance was 5–10 μmol photons m^−2^ s^−1^; differentiation frequencies reached 100% under white light and approximately 80% under green light, compared with 0–10% under red light, and the first 6 h constituted a critical light-sensitive interval. Glucose or sucrose supported nearly 100% differentiation even in darkness, whereas respiratory inhibition suppressed the response [13]. These findings suggest that light and metabolisable carbon both influence the energetic conditions that permit hormogonium differentiation.

Research on *Nostoc punctiforme* shows that hormogonia are specialised motile filaments controlled by the hybrid histidine kinase HrmK and a hierarchical SigJ-SigC/SigF cascade; time-resolved transcriptomics has defined the early developmental regulon [14,15,16]. These mechanisms should not be assigned directly to *N. sphaeroides*, but they provide specific candidates for investigation. Stage-resolved expression in *N. sphaeroides* shows that genes associated with pili and hormogonia decline during the transition, while selected genes involved in photosynthesis, carbon fixation, nitrogen metabolism, heterocyst differentiation and matrix-associated functions increase during later vegetative development [8]. Colony formation therefore appears to be a regulated developmental transition rather than passive aggregation. Transcriptomes sampled at 0, 2 and 72 h after transfer of hormogonia to 20 μmol photons m^−2^ s^−1^ identified 5653 differentially expressed genes (|log2 fold change| > 1; false discovery rate < 0.05) [8]. These are gene-level selection criteria, not mean pathway responses.

Nitrogen availability must be considered together with light during these transitions. In the model *N. punctiforme*, removal of combined nitrogen can initiate either hormogonium or heterocyst differentiation, reflecting alternative developmental responses to nitrogen starvation [17]. Direct evidence in *N. sphaeroides* CCNUC1 instead comes from a light-shift experiment: lowering irradiance from 20 to 5–10 μmol photons m^−2^ s^−1^ induced mature hormogonia within 24–48 h, whereas returning hormogonia to 20 μmol photons m^−2^ s^−1^ in nitrogen-free BG11 medium at 25 °C produced heterocyst-bearing vegetative filaments within 72 h [8]. This sequence connects dispersal with subsequent diazotrophic establishment under combined-nitrogen limitation, but does not establish nitrogen withdrawal alone as the trigger for hormogonium formation in *N. sphaeroides*.

Heterocysts add a further level of multicellular organisation. Vegetative cells perform oxygenic photosynthesis and supply reduced carbon, whereas heterocysts protect nitrogenase and export fixed nitrogen. In model heterocyst-forming cyanobacteria, fluorescent-tracer experiments demonstrate rapid intercellular exchange through septal junctions, and disruption of the junction component SepN impairs gated communication and survival under stress [18,19,20]. Whether the three-dimensional colony environment alters exchange rates, junction gating or heterocyst spacing in *N. sphaeroides* remains unknown. Measurements of intercellular metabolite transfer across colonies of different sizes would help relate filament-level division of labour to macrocolony physiology.

## 3. The Spherical Macrocolony as a Microbial Microenvironment

### 3.1. The Extracellular Matrix Contributes to Structure and Stress Tolerance

The extracellular polymeric substance (EPS) matrix gives *N. sphaeroides* its characteristic form, but its ecological roles extend beyond mechanical cohesion. Cyanobacterial extracellular glycans are chemically diverse, often anionic and capable of influencing adhesion, hydration, cation binding, diffusion and interactions among cells or species [21,22,23]. In *N. sphaeroides*, soluble, released and cell-associated polysaccharide fractions change during development: reducing sugars are relatively prominent in hormogonia, whereas sulfated polymers accumulate during the colony stage [24,25]. This temporal shift is consistent with a transition from dispersal to investment in a persistent extracellular matrix.

Several observations support protective roles for the matrix but do not establish causality. A homologue of the water-stress protein WspA has been detected in *N. sphaeroides*, although at lower abundance than in the more desiccation-tolerant *N. commune* [26]. Purified polysaccharide fractions show water-retention and antioxidant-associated properties in vitro [27,28]. These results make hydration and redox buffering plausible, but isolated polymers do not reproduce the spatial organisation of the intact matrix, its associated proteins, bound ions or diffusion pathways.

Evidence from other macroscopic *Nostoc* species also cautions against equating abundant matrix with desiccation tolerance. Removing EPS from *N. commune* strongly reduces photosynthetic recovery after drying, whereas *N. verrucosum* retains a substantial extracellular matrix and accumulates trehalose but remains sensitive to desiccation [29,30]. In *N. flagelliforme* and *N. commune*, periodic drying is associated with coordinated changes in matrix flexibility and rigidity [31]. Polymer chemistry, matrix proteins, architecture and hydration history, rather than EPS abundance alone, should therefore be examined in *N. sphaeroides*.

Clarifying these functions will require structural and physiological evidence. Targeted perturbations of glycosyltransferases, export systems or sulfation pathways should be combined with measurements of polymer composition, rheology, water potential, ion binding, diffusion and survival through repeated drying-rewetting cycles. Genetic complementation or addition of purified polymer could distinguish direct matrix roles from indirect effects on growth. Until such evidence is available, EPS is best described as a multifunctional adaptive matrix whose individual contributions remain unresolved.

### 3.2. Colony Size Balances Persistence with Resource Exchange

Mature colonies are physiologically layered. Microscopy and activity measurements distinguish outer, middle and inner regions, with greater photosynthetic activity near the surface and a liquid-rich core in large colonies [32]. Light and dissolved substrates enter through the surface, while inner filaments depend on diffusion through EPS and overlying biomass. As in phototrophic biofilms and microbial mats, this geometry is expected to produce gradients of irradiance, oxygen, pH, inorganic carbon and metabolites [33]. Direct microsensor maps are not yet available for *N. sphaeroides*, so the identity and magnitude of most gradients remain hypotheses rather than measurements.

Other spherical phototrophic systems provide useful methodological precedents. In *Nostoc parmelioides* colonies, measurements at 50 µm resolution related N_2_ fixation and photosynthesis to oxygen, chlorophyll a and light [34]. Combined oxygen and spectral scalar-irradiance microsensors identified a photic zone of less than 0.7 mm in an epilithic cyanobacterial biofilm [35], while measurements of photogranules showed approximately 90% attenuation of visible light within 600 µm and concentrated major activities in the outer approximately 500 µm [36]. These values should not be applied directly to *N. sphaeroides*, but they illustrate the spatial resolution and combination of measurements needed to examine colony gradients.

The physiological effects of colony size are evident in culture studies. Smaller colonies generally have higher maximum photosynthetic rates, greater light-use capacity, stronger affinity for dissolved inorganic carbon and faster growth than larger colonies. Large colonies may experience greater surface photoinhibition and slower recovery [37,38]. Outdoor cultures inoculated with 1–2 mm colonies also outperform cultures started with 3–4 mm colonies [39]. Nevertheless, a large hydrated sphere may persist better through disturbance and develop the mechanical properties associated with mature colonies. Colony size therefore links ecological persistence with constraints on resource exchange rather than serving only as a morphological descriptor. For example, colonies 0.08 cm in diameter had a relative growth rate approximately 2.3-fold that of 0.6 cm colonies [37]. In 2.5 m^2^ outdoor raceways, 1–2 mm and 3–4 mm inocula yielded approximately 5.2 and 0.25 g dry weight m^−2^ d^−1^, respectively, over 24 days [39].

Future models should integrate surface irradiance, pigment-dependent attenuation, EPS diffusivity, respiration, carbon-concentrating mechanisms and colony growth. Oxygen and pH microelectrodes, planar optodes, optical coherence imaging and stable-isotope tracers could then help determine whether inner regions are dormant reserves, metabolically specialised compartments or simply biomass limited by diffusion. Reporting colony size distributions together with dry mass is an essential first step towards comparability among studies.

## 4. The Cyanosphere as a Component of Colony Ecology

Environmental and commercial cultures are often non-axenic. The macrocolony contains vegetative filaments and heterocysts embedded in a hydrated EPS matrix, together with associated heterotrophic bacteria; these cellular and extracellular components should be distinguished when reconstructing the consortium [8,32,40]. The EPS matrix provides attachment sites and organic substrates for bacteria, while heterotrophs may remineralise nutrients, consume oxygen, supply vitamins, remove inhibitory compounds or compete with the cyanobacterium. In *N. sphaeroides*, different organic carbon sources alter both cyanobacterial metabolites and the composition of associated bacteria [40]. Although direct evidence remains limited, this finding is important because a cultivation treatment can affect both the cyanobacterium and its microbial neighbourhood.

In the carbon-source experiment of Zhang et al., associated bacterial genera included *Acinetobacter*, *Brevundimonas* and *Sphingobium* [40]. These taxa provide concrete candidates for isolation from authenticated *N. sphaeroides* colonies, but their detection and association with metabolic changes do not establish obligate mutualism or a universal core microbiome. Candidate partners should therefore be recovered from the colony system being tested, identified at strain level and examined individually for their effects on cyanobacterial growth, matrix formation and nutrient exchange.

Studies of other terrestrial cyanobacteria show that cyanosphere heterotrophs can accelerate cyanobacterial establishment and encode complementary nutrient functions [41]. In replicated bottom-up experiments, 108 consortia assembled from five model cyanobacteria and three freshwater inocula converged on a 25-species core microbiome enriched in micronutrient biosynthesis, metabolite transport and anoxygenic phototrophy [42]. A 2026 metagenomic survey of 56 terrestrial cyanobacterial cultures recovered 415 metagenome-assembled genomes and related cyanosphere composition to habitat, temperature and precipitation; predicted functions included nitrogen transformations and polysaccharide degradation [43]. These findings cannot be transferred species-for-species to *N. sphaeroides*, but they support the testable hypothesis that its colony microbiome contributes to nutrient retention, matrix turnover and stress buffering.

The cyanosphere also complicates experimental interpretation. Antibiotics, washing, serial transfer and organic carbon can alter the associated community without directly targeting *N. sphaeroides*. A treatment that increases colony growth may act through a bacterial partner; conversely, a beneficial partner may introduce variability or safety concerns. Future studies should compare axenic, conventional and reconstructed communities, sequence isolates and metagenomes, and use stable-isotope probing or paired metatranscriptomics to identify exchanges of carbon, nitrogen, sulfur and cofactors. A useful sequence of experiments would begin by adding a resident isolate, for example, a *Brevundimonas* or *Sphingobium* isolate recovered from the same colony stock, to an axenic *N. sphaeroides* culture. Cyanobacterium-only, bacterium-only and cell-free spent-medium controls would help distinguish growth promotion from metabolite carry-over or competition. Validated isolates could then be combined into replicated, defined communities examined across carbon sources and EPS perturbations. Such communities may provide more reproducible ecological experiments than either uncontrolled mixed cultures or strictly axenic cultures.

## 5. Environmental Factors Influence Photosynthetic Energy Balance

### 5.1. Visible Light Is Both a Developmental Cue and an Energy Supply

Light influences developmental state as well as photosynthesis. Low irradiance is associated with the dispersal phase, whereas increased light accompanies vegetative reconversion, greater photosynthetic investment, heterocyst development and colony formation [8,13]. Spectrum is also important: white and blue light can favour growth or phycobiliprotein accumulation, whereas red light may favour other biomass or pigment responses depending on dose and developmental stage [44]. Research across cyanobacteria shows that photoreceptors and redox-responsive regulators jointly influence chromatic acclimation, but the relevant photoreceptors have not been identified in *N. sphaeroides* [45].

Reported optimal photon fluxes cannot be reduced to a single value. Studies have examined different outcomes, including hormogonium differentiation, pigment accumulation, growth and colony maturation, using different optical paths, spectra, inoculum densities and strains [13,44,46]. Scalar irradiance within a culture or colony is the relevant exposure rather than incident light alone. Developmental history should also be reported because the same light dose may have different effects before and after vegetative reconversion. For pigment-oriented culture, Ma et al. reported favourable performance under white light at 90 or blue light at 30 μmol photons m^−2^ s^−1^ [44]. A separate suspension-culture study used 150 μmol photons m^−2^ s^−1^, 25 ± 2 °C, a 16 h light/8 h dark cycle and 50 rpm agitation, reaching 0.610 g dry weight L^−1^ after 40 days [46]. These examples describe different culture systems and endpoints, rather than a shared optimum.

### 5.2. Ultraviolet Responses Depend on Dose and Context

Ultraviolet B (UV-B) exposure can cause pigment loss, oxidative stress and photosynthetic injury in *N. sphaeroides*, while antioxidant systems and externally supplied protective compounds can lessen the damage [47]. Cyanobacteria also produce ultraviolet-screening compounds such as mycosporine-like amino acids [48,49]. These responses are consistent with stress at exposed paddy-field surfaces and during outdoor cultivation. For example, at a reported UV-B irradiance of 1 W m^−2^ for 5 h at 25 °C, maximum PSII photochemical efficiency (Fv/Fm) fell to 22% of its pretreatment value in non-acclimated colonies; colonies previously acclimated to low UV-B for 12 days retained 30% [50].

Under photosynthetically active radiation (PAR) of 20 μmol photons m^−2^ s^−1^, supplementation with 0.08 W m^−2^ biologically effective UV-B increased specific growth rate by 16% and dry biomass after 14 days by 30%, alongside increased photosystem I abundance, cyclic electron flow and D1 synthesis or repair [50]. The biologically weighted irradiance should be distinguished from unweighted UV measurements. With the same background PAR, ultraviolet A (UV-A) at 5, 10 and 15 W m^−2^ increased dry biomass after 16 days by 25%, 56% and 15%, respectively. At 10 W m^−2^ UV-A, the NADP pool and Rubisco activity were 1.8- and 2.3-fold the control values, respectively, and ATP content increased by 83% [51]. However, this UV-A irradiance reduced biomass by 9–23% when background PAR exceeded 50 μmol photons m^−2^ s^−1^ [51]. Proteomic analyses show coordinated changes in pigment synthesis, electron transfer, carbon-concentrating mechanisms, ribosomes, DNA repair, mycosporine-like amino acids and glutathione metabolism [9]. Thus, low ultraviolet exposure can act as an acclimatory cue when energy from visible light is limited.

The strongest causal evidence concerns the sensor kinase NsHik33 and the response regulator NsRpaB. Disruption of *nshik33* removes the growth benefit of low-dose UV-B, linking the pathway to photosystem gene regulation and increased photosynthetic capacity [10]. This work also establishes CRISPR-Cpf1 manipulation in the organism. The ecological conclusion is not that UV-B is generally beneficial, but that its effects depend on visible light, temperature, mixing, pigment state and colony size. Reactive oxygen production and photosystem damage predominate when exposure exceeds the capacity for repair and metabolic adjustment.

### 5.3. Electron Flow Is Closely Linked to Inorganic Carbon Acquisition

The *N. sphaeroides* CCNUC1 genome contains a chromosome of approximately 6.48 Mb, a 2.34 Mb megaplasmid and seven smaller plasmids [8]. It encodes multiple *psbA*-related loci and six flavodiiron proteins. In cyanobacteria, Flv1/Flv3 provide an alternative electron sink that protects photosystem I during fluctuating light, while Flv2/Flv4-related systems dissipate excitation around photosystem II [52,53]. The presence of several copies suggests possible adaptation to transitions between shade and sun, although their individual functions in *N. sphaeroides* have not been tested.

During reconversion from hormogonia to vegetative cells, transcripts associated with bicarbonate uptake, carboxysomes, Rubisco and Calvin–Benson–Bassham cycle enzymes increase [8]. Cyanobacterial carbon-concentrating mechanisms elevate CO_2_ around Rubisco by coordinating inorganic carbon transport with carboxysome chemistry [54]. Evidence from low-dose ultraviolet treatments further links cyclic electron flow and ATP/NADPH balance with carbonic anhydrase and Rubisco activities [9,51]. Analyses of whole-colony carbon use should therefore consider external diffusion, active bicarbonate uptake, photosynthetic demand and the recycling of respiratory CO_2_ within the colony.

The small RNA PsrR1 may provide an additional post-transcriptional layer. It responds to developmental stage in *N. sphaeroides*, and its expression is inversely related to that of predicted photosynthetic targets [8]. In model cyanobacteria, PsrR1 regulates photosystem transcripts within an RpaB-dependent light-acclimation network [55]. Direct validation of targets, measurements of RNA half-life and perturbation of PsrR1 are needed before an equivalent regulatory relationship can be proposed for *N. sphaeroides*.

### 5.4. Carbon, Nitrogen, Phosphorus and Calcium Jointly Affect Development

Exogenous organic carbon acts as both a substrate and a signal. Glucose or sucrose can substitute for light-derived energy during hormogonium differentiation, while different carbon sources alter pigments, metabolites and associated bacterial communities [13,40]. The effects are likely to depend on oxygen availability, contamination and cyanosphere composition. Organic carbon should therefore be examined as a community-level environmental factor rather than simply as an added carbon source.

Combined nitrogen reduces the requirement for energetically costly heterocyst differentiation but can also become inhibitory. At pH 8.3 ± 0.2 and 25 °C, the 96 h ammonium EC50 for relative growth rate was 1.105 mmol L^−1^. Exposure to 1 mmol L^−1^ NH4Cl for 96 h reduced photosynthetic rate, maximum PSII photochemical efficiency (Fv/Fm) and PSII activity; chlorophyll a synthesis was more sensitive than phycobiliprotein synthesis [56]. Exposure depends on nitrogen form, the rate of addition, pH-dependent free ammonia and diffusion within colonies. Phosphate produces a different response: after 16 days, 50 or 250 μmol L^−1^ phosphate supported 62–73% spherical colonies, compared with 10–15% at 0.5 or 5 μmol L^−1^ [57]. Field distributions also implicate phosphorus within a broader mineral and hydrological context [3,4].

Calcium also promotes growth and spherical-colony formation. After 18 days, the proportion of spherical microcolonies reached 88–93% at 0.25, 1 or 4 mmol L^−1^ Ca^2+^, compared with approximately 51% at 0.01 or 0.06 mmol L^−1^; the absolute growth rate based on dry weight was 23–30% higher than in the lower-calcium treatments [58]. Possible explanations for calcium-promoted colony morphogenesis include EPS cross-linking, adhesion, membrane signalling and ionic homeostasis, but their individual causal contributions have not been demonstrated directly. Experiments using chelators and ion substitution, together with measurements of free calcium, matrix-bound calcium, polymer rheology and gene expression, could distinguish structural from signalling roles.

### 5.5. Hydration, Temperature and Agrochemicals Shape Stress Tolerance

Temperature affects enzyme kinetics, gas solubility, respiration and membrane state, while drying concentrates salts and slows diffusion. Seasonal cultivation and colony physiology both show temperature dependence, but the cold- and heat-response mechanisms of *N. sphaeroides* are much less well understood than its ultraviolet response [32,59]. Evidence from other cyanobacteria suggests roles for membrane fluidity, sigma factors, chaperones and redox signalling, but these interpretations require species-specific tests [45].

Hydration should be examined as a dynamic condition. Water retention by EPS and the presence of WspA are consistent with buffering against water loss, but *N. sphaeroides* should not be assumed to share the extreme desiccation tolerance of *N. commune* or *N. flagelliforme* [26,27]. Experiments should measure survival, lag time, photosystem recovery, nitrogenase reactivation and mechanical properties across controlled drying rates and repeated cycles. The rate of rewetting may itself impose osmotic and mechanical stress.

**Figure 2:**
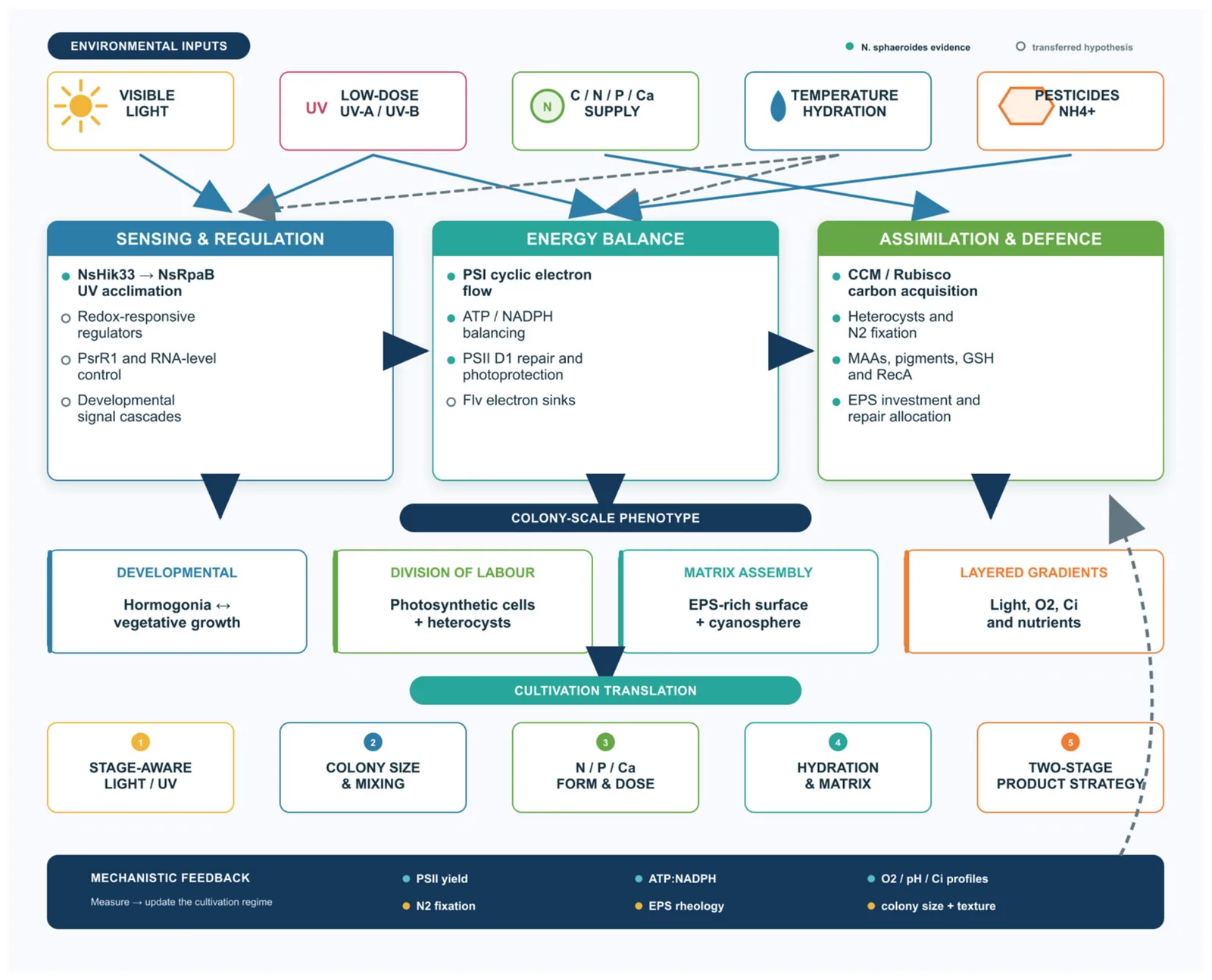
Conceptual relationships between environmental factors and colony development in *N. sphaeroides*. Light, low-dose ultraviolet radiation, nutrients, temperature, hydration, ammonium and pesticides influence sensing, photosynthetic energy balance, carbon and nitrogen assimilation, repair and stress responses. These processes affect developmental state, extracellular-matrix formation, the cyanosphere and gradients within colonies. Filled symbols and solid arrows indicate evidence obtained directly in *N. sphaeroides*; open symbols and dashed arrows indicate hypotheses based on model cyanobacteria. Abbreviations: ATP, adenosine triphosphate; C, carbon; Ca, calcium; CCM, carbon-concentrating mechanism; EPS, extracellular polymeric substances; Flv, flavodiiron protein; GSH, glutathione; MAAs, mycosporine-like amino acids; N, nitrogen; NADPH, reduced nicotinamide adenine dinucleotide phosphate; NH_4_^+^, ammonium; NsHik33, *N. sphaeroides* histidine kinase 33; NsRpaB, *N. sphaeroides* response regulator RpaB; O_2_, oxygen; P, phosphorus; PSI/PSII, photosystems I/II; PsrR1, photosynthesis-related small RNA 1; RecA, recombinase A; Rubisco, ribulose-1,5-bisphosphate carboxylase/oxygenase; UV-A/UV-B, ultraviolet A/B.

Paddy-field agrochemicals can reduce physiological resilience. Thiobencarb concentrations above 2 mg L^−1^ decreased phycobiliprotein contents, whereas 10 mg L^−1^ reduced biomass yield, protein content and photosynthetic rate and increased susceptibility to photoinhibition [60]. Other pesticides have compound- and dose-dependent effects: at 150 μmol L^−1^, butachlor and bensulfuron-methyl inhibited PSII and PSI, respectively, illustrating that equal molar exposure need not affect the same photosynthetic target [61]. EPS may initially bind chemicals and reduce exposure, but retention near cells could also delay their release. Experiments with chronic mixtures, sediment sorption and sequences of fertiliser and pesticide exposure would better represent paddy-field conditions than a single acute treatment. The relationships discussed in this section are summarised in Figure 2 and Table S1.

## 6. Genome Organisation and Regulatory Mechanisms

### 6.1. Genome Organisation May Contribute to Ecological Flexibility

The large chromosome, megaplasmid and seven smaller plasmids of strain CCNUC1 encode diverse regulatory and metabolic functions [8,62]. Accessory genes include candidates associated with anoxic metabolism, phosphonate use, gas vesicles and environmental responses. Such functions could differ between the surface and core of a stratified colony. However, genome size and annotation alone do not demonstrate ecological function, and expression measured from whole colonies averages across depths, cell types and associated bacteria.

Comparative long-read genomes, measurements of plasmid copy number, targeted plasmid curing and complementation are needed to assign function. A pan-genome including environmental isolates, commercial strains and principal laboratory strains would help determine whether ultraviolet acclimation, matrix pathways and nutrient responses are conserved or restricted to particular lineages. This distinction is especially important because the current species name includes considerable genome-wide divergence.

### 6.2. Testing Regulatory Hypotheses Derived from Omics Studies

Available evidence links sensory kinases and redox status with response regulators and small RNAs that may alter photosystem composition, electron flow, carbon and nitrogen assimilation, and developmental investment. NsHik33-NsRpaB is the first component of the ultraviolet response supported by targeted genetic evidence. PsrR1, flavodiiron proteins, *psbA* copies, bicarbonate transporters and EPS genes remain important candidates whose proposed roles are based on expression patterns or conserved functions rather than direct perturbation. Table S2 summarises the available evidence, proposed functions and research priorities for each candidate.

The CRISPR-Cpf1 method enables functional analysis, although redundancy is likely to complicate interpretation of single-gene knockouts. Multiplex editing, CRISPR interference, promoter replacement and inducible complementation should be combined with measurements extending beyond bulk optical density, including developmental timing, colony size distributions, internal light and gas gradients, photosystem kinetics, nitrogenase activity, matrix mechanics and recovery after fluctuating stress. Closely spaced time-course sampling is also important because sensing, vegetative reconversion, heterocyst patterning and matrix deposition occur on different timescales.

## 7. Implications of Environmental Adaptation for Cultivation

### 7.1. Cultivation Goals Should Distinguish Biomass Yield from Colony Quality

Dry biomass, rapid production of small colonies, large spherical colonies, pigment enrichment, EPS yield, selenium accumulation and mechanical firmness are distinct cultivation outcomes. Because enlargement may improve persistence and texture while reducing photosynthetic efficiency, conditions selected only for volumetric growth may not produce the desired colony phenotype. Studies should therefore define strain identity, developmental stage, colony size distribution, roundness, firmness, pigment and protein ranges, matrix composition and contaminant limits according to the intended outcome.

Developmental stage may also inform the organisation of culture conditions. Separate periods could favour hormogonia or small inocula, support photosynthetic growth and nitrogen assimilation, and then promote spherical form and matrix deposition. This approach reflects the natural life cycle and may help separate cultivation aims that cannot be achieved simultaneously.

### 7.2. Cultivation Conditions Should Account for Developmental Stage

Inocula should be described by size distribution rather than wet mass alone. The outdoor comparison of 1–2 mm and 3–4 mm inocula described in Section 3.2 provides a quantitative basis for testing small-colony starters [39]. Small colonies provide more photosynthetically active surface area, but mechanical fragmentation may damage filaments, alter associated bacterial communities and release dissolved organic carbon [37,39]. When fragmentation is used, a recovery period and monitoring of community composition are advisable. Imaging can be used to quantify diameter, circularity and breakage over time.

Light, nutrients and carbon should be considered together with developmental stage. Published light regimes include 5–10 μmol photons m^−2^ s^−1^ for maintaining the hormogonial phase, followed by a return to 20 μmol photons m^−2^ s^−1^ for vegetative reconversion [8], and white light at 90 or blue light at 30 μmol photons m^−2^ s^−1^ for pigment-oriented growth [44]. The UV-B and UV-A growth experiments summarised in Section 5.2 lasted 14 and 16 days, respectively, under low visible light; these experimental regimes require validation before transfer to larger cultures [50,51]. Reported morphogenesis-supporting treatments used 50 or 250 μmol L^−1^ phosphate and 0.25, 1 or 4 mmol L^−1^ calcium in separate experiments [57,58]. These concentrations provide starting points for strain-specific trials; they do not establish a jointly optimised medium. Ammonium delivery must account for the inhibitory response at 1 mmol L^−1^ NH4Cl, as well as pH and free ammonia [56]. The use of organic carbon also requires attention to oxygen availability and cyanosphere composition [40].

A two-stage pilot study at 300 L scale first grew biomass in nitrogen-free BG11 medium and then transferred colonies to selenium-supplemented, phosphorus-reduced, nitrogen-containing BG11 medium. The study reported approximately 0.5 g dry weight L^−1^ biomass and a colony hardness of approximately 3 N [63]. In 500 L outdoor cultures testing initial fresh-biomass densities of 5, 10 and 15 g L^−1^, the highest fresh-biomass productivity among the tested densities occurred at 10 g L^−1^ in summer and autumn (0.26 and 0.31 g L^−1^ d^−1^) and at 15 g L^−1^ in winter and spring (0.52 and 1.40 g L^−1^ d^−1^) [59]. Fresh- and dry-weight yields should be reported separately because colony hydration strongly influences their relationship. Future cultivation studies should therefore report incident and internal light, temperature, optical depth, colony size, mixing and respiration so that results can be compared across seasons and facilities.

### 7.3. Cyanosphere Composition Affects Reproducibility and Safety

A non-axenic colony is an ecological consortium, and both experimental and production cultures should document its associated community. Defined starter cultures, closed seed cultures, records of community composition and traceable harvest lots may reduce the risk that an uncontrolled associate becomes a source of variation. Whole-genome screening for toxin and antimicrobial-resistance genes should be combined with targeted toxin analysis, measurements of heavy metals and pesticides, microbial contaminant testing and assessment of storage stability. Cyanotoxins have been detected in some commercial cyanobacterial products, which demonstrates a general need for verification but does not show that authenticated *N. sphaeroides* produces these toxins [64,65].

Environmental sustainability also requires quantitative assessment. Nitrogen fixation may reduce inputs of combined nitrogen but has an energetic cost. Water recycling can reduce demand while increasing the concentrations of salts, metabolites and opportunistic microorganisms. Life-cycle and economic assessments should include lighting, mixing, temperature regulation, nutrients, dewatering, losses caused by contamination and useful co-products. Claims of sustainability should therefore be based on clearly defined and measured system boundaries rather than on the identity of the organism alone. Table S3 summarises environmental considerations and measurements relevant to cultivation.

## 8. Conclusions

*Nostoc sphaeroides* responds to variation in paddy-field conditions through a developmental sequence that connects motile hormogonia, differentiated filaments, nitrogen-fixing heterocysts and an EPS-rich spherical macrocolony. The sphere retains water, binds ions and hosts associated bacteria, but enlargement also reduces light penetration and the exchange of gases and nutrients. This balance helps explain why colony size is simultaneously an ecological trait, a physiological state and an important consideration in cultivation.

At the cellular level, light, ultraviolet radiation, carbon, nitrogen, phosphorus, calcium, temperature, hydration and agrochemicals influence photosynthetic energy balance, carbon acquisition, repair and development. The NsHik33-NsRpaB pathway demonstrates that these relationships can be tested genetically, yet most proposed colony functions and interactions with associated microorganisms remain inferential. The complete genome, multi-omics resources and emerging CRISPR capability make *N. sphaeroides* increasingly accessible to experimental study. Further progress will require combining causal genetics with spatial measurements of colony physiology and defined cyanosphere communities under fluctuating conditions. Careful attention to strain identity and a clear distinction between direct and inferred evidence will strengthen the value of *N. sphaeroides* as a model of cyanobacterial developmental multicellularity.

## Data Availability

All mentioned genes and proteins are included in the Supplementary Materials.

## Supplementary Materials

The following supporting information can be downloaded at: https://www.mdpi.com/article/10.3390/plants15182856/s1.

## Abbreviations

The following abbreviations are used in this manuscript.

**U**V	Ultraviolet radiation
**C**i	Inorganic carbon
**E**PS	Extracellular polymeric substance
**F**lv	Flavodiiron protein
**M**AAs	Mycosporine-like amino acids
**P**srR1	Photosynthesis-related small RNA 1
**N**sHik33	N. sphaeroides histidine kinase 33
**N**sRpaB	N. sphaeroides response regulator RpaB
**P**SI/PSII	Photosystems I/II
**N**ADPH	Reduced nicotinamide adenine dinucleotide phosphate
**U**V-A/UV-B	Ultraviolet A/B

## References

[1] Shang J.L., Xie Y.X., Shi L.Y., Diao S.R., Guan J.Y. (2025). Molecular mechanisms of *Nostoc flagelliforme* environmental adaptation: A comprehensive review. Plants.

[2] Shang J.L., Qiu G.W., Zhao L., Xu H.F., Cheng Y., Li Y., Zhang Z.C., Dai G.Z., Hou S., Yang C. (2026). Guanidine fuels rapid resurrection of desert cyanobacteria. Proc. Natl. Acad. Sci. USA.

[3] Qiu B., Liu J., Liu Z., Liu S. (2002). Distribution and ecology of the edible cyanobacterium Ge-Xian-Mi (*Nostoc*) in rice fields of Hefeng County in China. J. Appl. Phycol..

[4] Qin H., Lu J., Wang Z., Li D. (2013). The influence of soil and water physicochemical properties on the distribution of *Nostoc sphaeroides* (Cyanophyceae) in paddy fields and biochemical comparison with indoor cultured biomass. J. Appl. Phycol..

[5] Zhu S., Xu J., Adhikari B., Lv W., Chen H. (2023). *Nostoc sphaeroides* cyanobacteria: A review of its nutritional characteristics and processing technologies. Crit. Rev. Food Sci. Nutr..

[6] Li F., Liu R., Qin S., Deng Z., Li W. (2025). Progress in culture technology and active substance research on *Nostoc sphaeroides* Kützing. J. Sci. Food Agric..

[7] Tauqir M., Sohail H., Xu J., Tareen M.B.K., Ismail M.A., Niaz N., Chauhdari T., Taj M.I., Chen G. (2026). Metabolic pathways and omics approaches in *Nostoc sphaeroides*: A roadmap for enhancing functional foods and health-related applications. Food Nutr. Health.

[8] Chen Z., Shang J.L., Hou S., Li T., Li Q., Yang Y.W., Hess W.R., Qiu B.S. (2021). Genomic and transcriptomic insights into the habitat adaptation of the diazotrophic paddy-field cyanobacterium *Nostoc sphaeroides*. Environ. Microbiol..

[9] Chen Z., Wu X., Liu Z., He Z., Yue H.H., Li F.F., Xu K., Shao H.C., Li W.Z., Chen X.W. (2025). Proteomic insight into growth and defense strategies under low ultraviolet-B acclimation in the cyanobacterium *Nostoc sphaeroides*. J. Photochem. Photobiol. B Biol..

[10] Chen Z., He Z., Luo W.X., Liu Z., Xu H.F., Yue H.H., Li F.F., Li W.Z., Chen C., Zhang S. (2026). Histidine kinase Hik33 plays a crucial role in UV-B-induced photosynthetic acclimation in cyanobacteria. Plant Physiol..

[11] Huo D., Gan N., Geng R., Cao Q., Song L., Yu G., Li R. (2021). Genome evolution of filamentous cyanobacterium *Nostoc* species: From facultative symbiosis to free living. Microorganisms.

[12] Jain C., Rodriguez-R L.M., Phillippy A.M., Konstantinidis K.T., Aluru S. (2018). High throughput ANI analysis of 90K prokaryotic genomes reveals clear species boundaries. Nat. Commun..

[13] Li D.H., Chen L.Z., Li G.B., Wang G.H., Song L.R., Liu Y.D. (2005). Photoregulated or energy dependent process of hormogonia differentiation in *Nostoc sphaeroides* Kützing (Cyanobacterium). J. Integr. Plant Biol..

[14] Gonzalez A., Riley K.W., Harwood T.V., Zuniga E.G., Risser D.D. (2019). A tripartite, hierarchical sigma factor cascade promotes hormogonium development in the filamentous cyanobacterium *Nostoc punctiforme*. mSphere.

[15] Harwood T.V., Risser D.D. (2021). The primary transcriptome of hormogonia from a filamentous cyanobacterium defined by cappable-seq. Microbiology.

[16] Risser D.D. (2023). Hormogonium development and motility in filamentous cyanobacteria. Appl. Environ. Microbiol..

[17] Christman H.D., Campbell E.L., Meeks J.C. (2011). Global transcription profiles of the nitrogen stress response resulting in heterocyst or hormogonium development in *Nostoc punctiforme*. J. Bacteriol..

[18] Nürnberg D.J., Mariscal V., Bornikoel J., Nieves-Morión M., Krauß N., Herrero A., Maldener I., Flores E., Mullineaux C.W. (2015). Intercellular diffusion of a fluorescent sucrose analog via the septal junctions in a filamentous cyanobacterium. mBio.

[19] Kieninger A.K., Maldener I. (2021). Cell-cell communication through septal junctions in filamentous cyanobacteria. Curr. Opin. Microbiol..

[20] Kieninger A.K., Tokarz P., Janović A., Pilhofer M., Weiss G.L., Maldener I. (2022). SepN is a septal junction component required for gated cell-cell communication in the filamentous cyanobacterium *Nostoc*. Nat. Commun..

[21] De Philippis R., Vincenzini M. (1998). Exocellular polysaccharides from cyanobacteria and their possible applications. FEMS Microbiol. Rev..

[22] Pereira S., Zille A., Micheletti E., Moradas-Ferreira P., De Philippis R., Tamagnini P. (2009). Complexity of cyanobacterial exopolysaccharides: Composition, structures, inducing factors and putative genes involved in their biosynthesis and assembly. FEMS Microbiol. Rev..

[23] Kehr J.C., Dittmann E. (2015). Biosynthesis and function of extracellular glycans in cyanobacteria. Life.

[24] Li D.H., Xing W., Li G.B., Liu Y.D. (2009). Cytochemical changes in the developmental process of *Nostoc sphaeroides* (cyanobacterium). J. Appl. Phycol..

[25] Zhong G., Pan W., Huang Z., Guo K., Hu J., Liu P., Chen S., Wang Y., Ai L., Huang Z. (2021). Physicochemical and geroprotective comparison of *Nostoc sphaeroides* polysaccharides across colony growth stages and with derived oligosaccharides. J. Appl. Phycol..

[26] Ai Y., Yang Y., Qiu B., Gao X. (2014). Unique WspA protein from terrestrial macroscopic cyanobacteria can confer resistance to osmotic stress in transgenic plants. World J. Microbiol. Biotechnol..

[27] Li H., Su L., Chen S., Zhao L., Wang H., Ding F., Chen H., Shi R., Wang Y., Huang Z. (2018). Physicochemical characterization and functional analysis of the polysaccharide from the edible microalga *Nostoc sphaeroides*. Molecules.

[28] Liu Y., Su P., Xu J., Chen S., Zhang J., Zhou S., Wang Y., Tang Q., Wang Y. (2018). Structural characterization of a bioactive water-soluble heteropolysaccharide from *Nostoc sphaeroids* Kutz. Carbohydr. Polym..

[29] Tamaru Y., Takani Y., Yoshida T., Sakamoto T. (2005). Crucial role of extracellular polysaccharides in desiccation and freezing tolerance in the terrestrial cyanobacterium *Nostoc commune*. Appl. Environ. Microbiol..

[30] Sakamoto T., Kumihashi K., Kunita S., Masaura T., Inoue-Sakamoto K., Yamaguchi M. (2011). The extracellular-matrix-retaining cyanobacterium *Nostoc verrucosum* accumulates trehalose but is sensitive to desiccation. FEMS Microbiol. Ecol..

[31] Liu W., Cui L., Xu H., Zhu Z., Gao X. (2017). Flexibility-rigidity coordination of the dense exopolysaccharide matrix in terrestrial cyanobacteria acclimated to periodic desiccation. Appl. Environ. Microbiol..

[32] Deng Z., Hu Q., Lu F., Liu G., Hu Z. (2008). Colony development and physiological characterization of the edible blue-green alga, *Nostoc sphaeroides* (Nostocaceae, Cyanophyta). Prog. Nat. Sci..

[33] Stal L.J., Bolhuis H., Cretoiu M.S. (2019). Phototrophic marine benthic microbiomes: The ecophysiology of these biological entities. Environ. Microbiol..

[34] Dodds W.K. (1989). Microscale vertical profiles of N2 fixation, photosynthesis, O_2_, chlorophyll a, and light in a cyanobacterial assemblage. Appl. Environ. Microbiol..

[35] Kühl M., Glud R.N., Ploug H., Ramsing N.B. (1996). Microenvironmental control of photosynthesis and photosynthesis-coupled respiration in an epilithic cyanobacterial biofilm. J. Phycol..

[36] Trebuch L.M., Bourceau O.M., Vaessen S.M.F., Neu T.R., Janssen M., de Beer D., Vet L.E.M., Wijffels R.H., Fernandes T.V. (2023). High resolution functional analysis and community structure of photogranules. ISME J..

[37] Li Y., Gao K. (2004). Photosynthetic physiology and growth as a function of colony size in the cyanobacterium *Nostoc sphaeroides*. Eur. J. Phycol..

[38] Gao K., Ai H. (2004). Relationship of growth and photosynthesis with colony size in an edible cyanobacterium, Ge-Xian-Mi *Nostoc* (Cyanophyceae). J. Phycol..

[39] Deng Z., Yan C., Lu F., Hu Q., Hu Z. (2008). Growth kinetics of 1–2 mm and 3–4 mm colonies of *Nostoc sphaeroides* (Cyanophyta) in outdoor culture. Biotechnol. Lett..

[40] Zhang J., Wang Q., Gong Q., Gao X. (2024). Symbiotic bacteria diversity and metabolome analyses of *Nostoc sphaeroides* grown under different organic carbon sources. Algal Res..

[41] Nelson C., Garcia-Pichel F. (2021). Beneficial cyanosphere heterotrophs accelerate establishment of cyanobacterial biocrust. Appl. Environ. Microbiol..

[42] Kust A., Zorz J., Cruañas Paniker C., Bouma-Gregson K., Krishnappa N., Liu W., Banfield J.F., Diamond S. (2025). Model cyanobacterial consortia reveal a consistent core microbiome independent of inoculation source or cyanobacterial host species. ISME J..

[43] Palmer B., Couradeau E.M., Johansen J.R., Kurbessoian T., Ortega Carranza J., Stajich J.E., Ward R., Pietrasiak N. (2026). Unraveling the diversity and functional potential of cyanosphere microbiomes assembled from terrestrial cyanobacteria. Appl. Environ. Microbiol..

[44] Ma R., Lu F., Bi Y., Hu Z. (2015). Effects of light intensity and quality on phycobiliprotein accumulation in the cyanobacterium *Nostoc sphaeroides* Kützing. Biotechnol. Lett..

[45] Rachedi R., Foglino M., Latifi A. (2020). Stress signaling in cyanobacteria: A mechanistic overview. Life.

[46] Arguelles E.D.L.R. (2025). Physiological characterization and morphogenesis of an edible cyanobacterium, *Nostoc sphaeroides* Kützing, in liquid suspension culture. J. Fish. Environ..

[47] Wang G., Hu C., Li D., Zhang D., Li X., Chen K., Liu Y. (2007). The response of antioxidant systems in *Nostoc sphaeroides* against UV-B radiation and the protective effects of exogenous antioxidants. Adv. Space Res..

[48] Sinha R.P., Hader D.P. (2008). UV-protectants in cyanobacteria. Plant Sci..

[49] Shang J.L., Zhang Z.C., Yin X.Y., Chen M., Hao F.H., Wang K., Feng J.L., Xu H.F., Yin Y.C., Tang H.R. (2018). UV-B induced biosynthesis of a novel sunscreen compound in solar radiation and desiccation tolerant cyanobacteria. Environ. Microbiol..

[50] Chen Z., Jiang H.B., Gao K., Qiu B.S. (2020). Acclimation to low ultraviolet-B radiation increases photosystem I abundance and cyclic electron transfer with enhanced photosynthesis and growth in the cyanobacterium *Nostoc sphaeroides*. Environ. Microbiol..

[51] Chen Z., Yuan Z.W., Luo W.X., Wu X., Pan J.L., Yin Y.Q., Shao H.C., Xu K., Li W.Z., Hu Y.L. (2024). UV-A radiation increases biomass yield by enhancing energy flow and carbon assimilation in the edible cyanobacterium *Nostoc sphaeroides*. Appl. Environ. Microbiol..

[52] Allahverdiyeva Y., Mustila H., Ermakova M., Bersanini L., Richaud P., Ajlani G., Battchikova N., Cournac L., Aro E.M. (2013). Flavodiiron proteins Flv1 and Flv3 enable cyanobacterial growth and photosynthesis under fluctuating light. Proc. Natl. Acad. Sci. USA.

[53] Bersanini L., Battchikova N., Jokel M., Rehman A.U., Vass I., Allahverdiyeva Y., Aro E.M. (2014). Flavodiiron Protein Flv2/Flv4-Related Photoprotective Mechanism Dissipates Excitation Pressure of PSII in Cooperation with Phycobilisomes in Cyanobacteria. Plant Physiol..

[54] Long B.M., Rae B.D., Rolland V., Forster B., Price G.D. (2016). Cyanobacterial CO_2_-concentrating mechanism components: Function and prospects for plant metabolic engineering. Curr. Opin. Plant Biol..

[55] Georg J., Dienst D., Schürgers N., Wallner T., Kopp D., Stazic D., Kuchmina E., Klähn S., Lokstein H., Hess W.R. (2014). The small regulatory RNA PsrR1 controls photosynthetic functions in cyanobacteria. Plant Cell.

[56] Dai G., Deblois C.P., Liu S., Juneau P., Qiu B. (2008). Differential sensitivity of five cyanobacterial strains to ammonium toxicity and its inhibitory mechanism on the photosynthesis of rice-field cyanobacterium Ge-Xian-Mi (*Nostoc*). Aquat. Toxicol..

[57] Chen Z., Chen S., Lu G., Chen X. (2012). Phosphorus limitation for the colony formation, growth and photosynthesis of an edible cyanobacterium, *Nostoc sphaeroides*. Biotechnol. Lett..

[58] Chen Z., Li W.Z., Chen J.Y., Yuan Z.W., Chen X.W. (2021). Effects of calcium ion on the colony formation, growth, and photosynthesis in the edible cyanobacterium *Nostoc sphaeroides*. J. Appl. Phycol..

[59] Wang B., Deng Z. (2025). Effects of initial density on biochemical composition and texture characteristics of *Nostoc sphaeroides* under long-term outdoor cultivation. Processes.

[60] Xia J. (2005). Response of growth, photosynthesis and photoinhibition of the edible cyanobacterium *Nostoc sphaeroides* colonies to thiobencarb herbicide. Chemosphere.

[61] Chen Z., Juneau P., Qiu B. (2007). Effects of three pesticides on the growth, photosynthesis and photoinhibition of the edible cyanobacterium Ge-Xian-Mi (*Nostoc*). Aquat. Toxicol..

[62] Shang J.L., Chen M., Hou S., Li T., Yang Y.W., Li Q., Jiang H.B., Dai G.Z., Zhang Z.C., Hess W.R. (2019). Genomic and transcriptomic insights into the survival of the subaerial cyanobacterium *Nostoc flagelliforme* in arid and exposed habitats. Environ. Microbiol..

[63] Hu J., Liu P., Wang Q., Nie X., Tan J., Shu J., Mai J., Cao Y., Zou Y., Huang Z. (2025). Pilot-scale production of selenium-enriched *Nostoc sphaeroides* colonies and polysaccharides using a two-phase cultivation strategy. Bioresour. Technol..

[64] Heussner A.H., Mazija L., Fastner J., Dietrich D.R. (2012). Toxin content and cytotoxicity of algal dietary supplements. Toxicol. Appl. Pharmacol..

[65] Vichi S., Lavorini P., Funari E., Scardala S., Testai E. (2012). Contamination by *Microcystis* and microcystins of blue-green algae food supplements. Food Chem. Toxicol..

